# Carbon Graphitization: Towards Greener Alternatives to Develop Nanomaterials for Targeted Drug Delivery

**DOI:** 10.3390/biomedicines10061320

**Published:** 2022-06-04

**Authors:** Davide Marin, Silvia Marchesan

**Affiliations:** Chemical and Pharmaceutical Sciences Department, University of Trieste, 34127 Trieste, Italy; davide.marin@studenti.units.it

**Keywords:** carbon nanomaterials, carbon nanotubes, graphene, carbon dots, targeted delivery, graphitization, drug delivery, waste, sustainability, cancer

## Abstract

Carbon nanomaterials have attracted great interest for their unique physico-chemical properties for various applications, including medicine and, in particular, drug delivery, to solve the most challenging unmet clinical needs. Graphitization is a process that has become very popular for their production or modification. However, traditional conditions are energy-demanding; thus, recent efforts have been devoted to the development of greener routes that require lower temperatures or that use waste or byproducts as a carbon source in order to be more sustainable. In this concise review, we analyze the progress made in the last five years in this area, as well as in their development as drug delivery agents, focusing on active targeting, and conclude with a perspective on the future of the field.

## 1. Introduction

**Figure 1:**
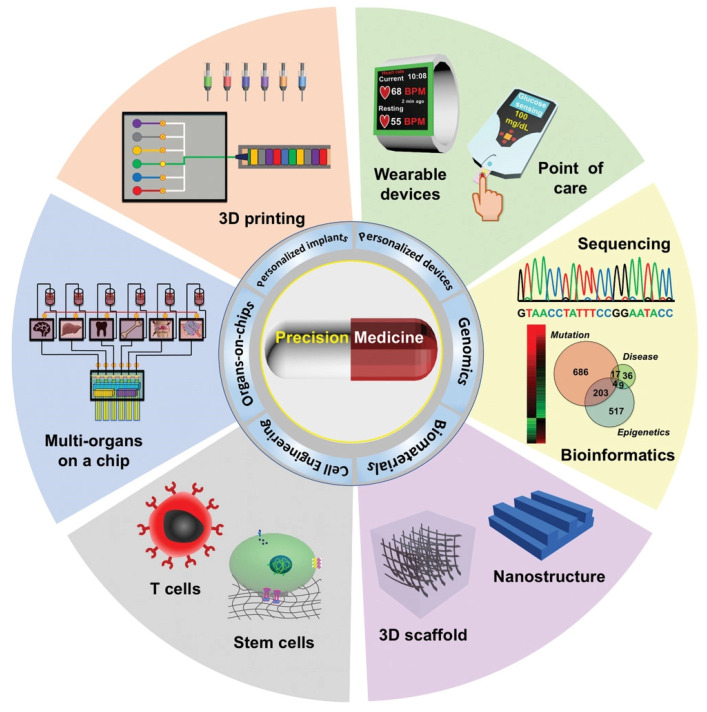
Various approaches to enable precision medicine. Reproduced from [9] under a Creative Commons licence.

There are high expectations for the next generation of medicines as we move towards personalized therapy (Figure 1) with targeted delivery for lower side effects [1]. Nanomaterials are well-positioned for a qualitative leap in addressing the most demanding, unsolved issues in medicine, thanks to their innovative properties [2] that enable multimodal imaging [3], combined diagnostics and therapy in theranostics [4], personalized drug delivery at the pathological site [5], and so on. In particular, carbon nanomaterials (CNMs) display unique physico-chemical properties, thanks to their graphitic nature, for unparalleled applications in drug delivery; however, critical steps in terms of thorough biosafety assessments and regulatory requirements must be taken to enable their clinical implementation [6,7]. Graphitization is a popular process not only to produce but also to modify CNMs and enhance their physico-chemical properties, but it is generally an energy-intensive step for which greener alternatives are continuously sought [8]. In this concise review, we will analyze the last 5 years of progress in the development of more sustainable ways to graphitize CNMs and their applications in drug delivery for the most challenging unmet needs in medicine and conclude with a perspective on the future of this exciting area of research.

## 2. Carbon Nanomaterials (CNMs)

**Figure 2:**
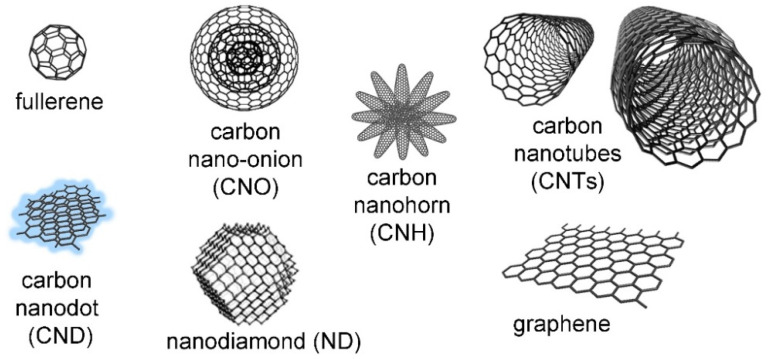
Carbon nanomaterials (not to scale), reproduced from [25]. The nano-onion is reproduced from [26]; copyright © 2022 with permission from Elsevier.

CNMs come in different sizes, morphology, curvature, and reactivity. The diverse members of this family (Figure 2) have been discovered through the years since the 1960s [10], with new ones continuously being produced, thanks to top-down or bottom-up approaches [11,12,13]. A common feature is that they are mainly composed of carbon atoms that are typically sp^2^ hybridized and that are arranged in a honeycomb lattice [14]. However, exceptions exist, such as nanodiamonds that feature a large portion of sp^3^ hybridization, as the name suggests [15]. A simple way to understand nanocarbon structure is to consider the two-dimensional graphene sheets [16] as a common building block so that, depending on how it is folded, they will yield different CNMs [17]. For example, fullerenes are soccer-ball-shaped [18], and carbon nano-onions (CNOs) are concentric fullerenes [19]. Carbon nanotubes (CNTs) are tubular in shape, and they occur as single-walled [20] or multi-walled [21]. Nanohorns (CNHs) consist of clusters of nanocones [22], while nanodots (CNDs) have been attracting increasing attention, thanks to their ultra-small size (<10 nm) and luminescence [23]. Graphene nanoribbons (GNRs) display yet another morphology, as the name suggests, and they are highly researched for the possibility they offer to fine-tune their electronic properties and, in particular, the bandgap through the modulation of structural features, such as width, orientation, backbone and edge structure, heteroatom doping, and, in general, their quality [24].

In spite of the large number of scientific studies on CNMs, often, it is difficult to anticipate which type is the best performer for specific applications, and even more so in the case of biological uses where there is a high degree of biomolecular complexity [27,28,29,30]. Interactions between CNMs and biomolecules, such as proteins [31] and DNA [32], are very relevant in this regard for their key roles in the determination of the dynamic corona on their surface [33], which, in turn, affects their fate in vivo [34], spanning their biodistribution [35] to immune response [36,37] and biodegradation [38,39].

All CNMs share many properties, such as low density, high mechanical resistance, good electronic conductivity, and the ability to undergo various types of chemical functionalization to fine-tune their properties [40], especially for clinical applications [41]. Unsurprisingly, many reviews exist for their successful use in biomedicine [42,43] and, in particular, oncology [44], theranostics [45], and bioimaging [46]. In drug delivery, they have been mainly investigated for cancer therapy and, more recently, also for atherosclerosis [47], microbial infections [48,49], and neurological disorders [50].

Nevertheless, concerns exist over their biosafety profile [51] and immunogenicity [52]; the relevant studies are further complicated by the high heterogeneity of this class of nanomaterials [53]. Various committees are thus tackling the development of unified standards to assist with their correct classification [54] and a general framework to enable their reliable risk assessment for the responsible advancement of nanotechnology [55]. This is not an easy task; however, international standards organizations (e.g., ISO (ISO/TC 229 Nanotechnologies) and IEC (IEC/TC 113 Nanotechnology for electrotechnical products and systems)) have already released various standards for graphene. Countries such as the USA, the UK, Korea, Japan, and especially China play an active role in graphene research, and a number of new standards are being continuously issued, especially in China, to assist the industrialization of graphene-related materials (Table 1) [56].

To speed up research on CNMs, cutting-edge in silico approaches such as machine-learning [57] are key enablers to fully exploit large datasets for which innovative methods for efficient management are also crucial [58]. Another critical step for the wide implementation of CNMs pertains to cost-effective large-scale production [59], ideally in a sustainable manner, in light of the current climate crisis and the urgent need to lower our impact and preserve the environment. The next section will thus focus on the recent efforts to find graphitization routes that are less energy-intensive than traditional methods.

## 3. The Graphitization Process

Graphitization is the conversion of amorphous carbon into graphitic carbon. This process requires a drastic rearrangement of chemical bonds to form a layered structure made of condensed six-membered sp^2^ carbon rings. For this reason, graphitization is a very energy-demanding process, and it is typically performed by heating petroleum coke up to 3000 °C in furnaces to produce synthetic graphite at the industrial level. The introduction of metal catalysts allows us to operate at lower temperatures [60], but metal residues are difficult to separate from the product.

Crystallization is also referred to the transformation of non-graphitic carbon into well-organized graphitic CNMs [61] such as fullerenes [62], graphene [63], CNTs [64,65], CNHs [66], CNOs [67], graphitic quantum dots (GQDs) [68], and graphitized carbon black (GCB). There are a lot of ways currently used to prepare this kind of structure, such as arc-discharge, chemical vapor deposition (CVD), laser ablation [69], pyrolysis, and thermal annealing [70], but all these methods involve the use of high amounts of energy and non-sustainable carbon sources. This limits the application of these materials on a large scale in technological and biomedical fields; therefore, the finding of green and economical routes to high-quality CNMs is a crucial challenge today.

## 4. Greener Alternatives to Traditional Graphitization for CNM Production

In recent years, great efforts have been devoted to finding green and economical ways to prepare CNMs in order to make them more sustainable and widely accessible. The literature in this field is in continuous evolution as many research groups are working hard to provide a solution to the problem. Therefore, in this review, we will focus on the most recent advances organized by the type of carbon source, in particular: waste, natural sources, atmospheric particulate, graphite or coal-derived products, and CO_2_. Table 2 reports the type of CNM, the green process employed, the carbon source used, and the investigated application for the most recent works discussing the green preparation of CNMs.

### 4.1. CNMs from Waste

The valorization of waste is currently a popular topic, especially for organic and plastic waste, which could be potentially used as carbon sources to produce CNMs in a greener and more economical way [142]. Examples were reported for both classes of waste.

As organic waste, waste frying oil has been used as a precursor for the preparation of CNOs with high electrochemical performance through traditional flame pyrolysis [72]. Microwave-assisted pyrolysis of fish scales produced oxidized CNOs with good light emission properties to fabricate a blue LED [73]. The nickel-catalyzed carbonization of rice husk, followed by chemical activation, was used to produce porous CNOs with a high degree of graphitization and excellent electrochemical properties to make supercapacitors [82]. Even barbeque grease has been used as a carbon source to produce CNTs by chemical vapor deposition [98].

As plastic waste, polymers such as polyethylene terephthalate (PET), polypropylene (PP), and polyethylene (PE) from plastic objects have been used to prepare CNMs with various methods. The catalytic thermal decomposition of PET from plastic bottles led to various CNMs, including fullerenes and CNTs, depending on the reaction conditions [143]. Graphene nanosheets suitable for energy applications were obtained by a combination of heating treatments and pyrolysis [138]. Fullerenes [84] and graphene [136,137] were prepared by thermal decomposition for industrial dye removal, and CNTs were prepared by catalyzed pyrolysis for use as lubricants [119]. Finally, numerous recent examples of CNTs prepared from plastic waste employed CVD with a 2-step reactor [101,102,103,104,105,106,107,108,109,110,111,112,113,114,115,116,117]. In brief, the typical reactor consists of two chambers: in the first chamber, plastic sources are pyrolyzed to produce hydrocarbon gases, which then act as precursors for the catalytic decomposition and deposition that happen in the second chamber. In some cases, there is a condensation step after the first chamber to allow further re-cracking of the heaviest hydrocarbon to light gases in order to increase the yield of the process. Table 3 summarizes the main characteristics of the recently reported processes with the best results obtained in terms of CNT quality. A similar process was employed to produce graphene sheets with a high graphitization degree used to make electrodes from various types of plastic waste [139].

### 4.2. CNMs from Sustainable Natural Sources

Natural sources are becoming very popular for the preparation of CNMs, especially for GQDs. For example, the hydrothermal treatment of fruit [123], cotton [124], starch [125], or lemon juice [126] leads to GQDs with excellent and tunable optical properties, used for bioimaging or heavy-metal sensing. To the same end, citric acid [127] and casein [128] can be used through pyrolysis, grape seeds extract through microwave treatment [131], or salicylic acid by means of UV-induced polymerization [130]. The hydrothermal carbonization of citric acid produced CNOs [81], while the pyrolysis of flaxseed oil led to CNOs with good optical properties for photocatalysis and Al(III) detection [71].

Another popular approach is based on using natural carbon precursors for the CVD synthesis of CNTs because it is one of the best methods to control CNT morphology. Plant extracts were used for a metal-free CVD approach towards CNTs of different types and graphitization degrees, depending on the temperature and the plant used [99]. Coconut and olive oil acted as a carbon source for spray pyrolysis (that is a variant of CVD), with the latter producing the best CNTs in terms of the level of graphitization [118].

### 4.3. Atmospheric Particulate as Carbon Source for CNMs

Great efforts are being devoted to the valorization of carbon soot—which is normally considered a pollutant—as a source for functional CNMs, and indeed, it was found to contain CNMs [144]. For this purpose, there are also examples of flying ashes used as carbon sources and/or catalysts for the CVD growth of CNTs as they contain alumina, silica, iron oxides, and other metal compounds that can catalyze CNT growth [100,145].

Recent works reported the isolation of CNOs from diesel soot by a Soxhlet purification of the as-collected particulate, to be used for bioimaging and Cr(VI) sensing [74], strain sensing [75], and dye removal [76]. Graphene nanosheets for the photocatalytic removal of dyes were also obtained by Soxhlet purification of diesel pollutant soot [132,133]. However, this kind of purification involves organic solvents, such as acetone, toluene, or petroleum ether, that are best avoided.

Candle soot is another good and costless source of CNMs, demonstrated to obtain CNOs with good photothermal properties for cancer therapy [79]. Finally, from a candle doped with iron acetylacetonate, magnetic CNOs were obtained and used for the efficient removal of bisphenol A from water [80].

### 4.4. Greener Routes to Prepare CNMs from Graphite- or Coal-Based Products

Even if graphite- or coal-based products are not green starting materials, it is important to develop low energy-demanding processes that can be useful in combination with recent advances to obtain synthetic graphite in greener ways [146].

Electrochemical treatment of a graphite rod produced GQDs that were marked with technetium-99m for radioimaging [120], while gamma irradiation of graphite produced GQDs that were suitable for photodynamic therapy after functionalization with urea [122]. Electrochemical treatment of wood charcoal yielded GQDs that could be used as a peroxidase mimic [121]. GQDs could also be obtained by mild oxidation of coal tar pitch with hydrogen peroxide [129]. A simple mechanical process such as ball milling produced CNOs starting from graphite powder [83]. Reduced graphene oxide (rGO) was obtained from recycled charcoal by chemical graphitization and oxidation, followed by green exfoliation by sonication and reduction with ascorbic acid [119]. In a similar way, rGO for Ni(II) removal was obtained using *Tecoma stans* leaf extract to reduce GO [135]. Finally, in a recent work, a catalytic reduction process to obtain a very high-quality rGO was reported [134]. In this case, the use of a Fischer–Tropsch catalyst in the absence of hydrogen allowed it to operate at the very low temperature of 250 °C, with oxygen as the only byproduct.

### 4.5. CO_2_ as Carbon Source to Produce CNMs

CO_2_ is the main greenhouse gas that affects global warming, with the removal and recycling of CO_2_ produced by human activities being one of the biggest challenges for scientists. For example, in a recent work, a high-quality synthetic graphite was produced by the chemical reduction of CO_2_ with LiAlH_4_ [146].

CO_2_ has been envisaged as a carbon source for the production of CNMs. The first chemical synthesis of CNTs from CO_2_ was reported by Motiei et al. [147]. They reacted Mg with supercritical carbon dioxide to obtain MgO and carbon, with a small yield of fullerenes and CNTs with good crystallinity. CO_2_ was also used as the only precursor gas [148,149] or as a co-precursor [150] for the production of CNTs and graphene by CVD. Recently, the synthesis of bilayered graphene with a very high graphitization degree was reported, using CO_2_ as the carbon source for CVD [140].

An approach that is attracting great attention is the electrolysis of CO_2_ in molten salt, introduced by The Licht C2CNT (carbon dioxide to CNT) group [151]. Various CNMs were recently obtained with this method, including CNOs, CNTs, graphene, carbon nanofibers, and other more exotic structures termed carbon nanodragons or nanoflowers [77,78,85,86,87,88,89,90,91,92,93,94,95,96,97,141]. Briefly, experiments were carried out in an alumina crucible containing an electrolyte (typically lithium carbonate), which was heated up to melting. Then, electrodes were immersed in the electrolyte, and a constant current was applied across the electrodes. Carbon products accumulated at the cathode, and they could be easily recovered, while oxygen was produced as a byproduct. A transition metal-based nucleation agent could be added to the cathode or in the molten salt as a catalyst or could originate from the erosion of the anode during the process. This technique is almost costless (excluding the electrodes), and it is very versatile as a large variety of CNMs can be obtained under varying reaction parameters, such as electrode composition, nucleation agent, electrolysis time, and current density [78]. For example, the presence of metal nucleation agents (typically iron, nickel, or chromium) usually leads to the formation of CNTs, while in their absence, CNOs [77] or graphene [141] are produced. The use of labeled ^13^CO_2_ revealed that carbonate was transformed into CNMs but was continuously renewed by atmospheric CO_2_, which was the real carbon source [152].

## 5. Applications of CNMs for Targeted Drug Delivery

CNMs are very interesting as drug delivery agents because of their high versatility. They are typically hydrophobic and well uptaken by cells, and they can also cross biological membranes such as the blood–brain barrier (BBB) [153,154]. In addition, as nanomaterials, passive targeting is possible due to the enhanced permeability retention (EPR) effect [155]. However, this approach has proved to be very effective in experimental studies on animal models but demonstrated limited efficacy in humans [156], with active targeting and personalized medicine holding promise to enable significant progress in the clinic.

CNMs can be functionalized covalently or non-covalently using organic chemistry with a plethora of molecules and biomolecules, and combined approaches are possible. For example, it is possible to append a drug and a targeting agent or multiple drugs for combined therapy or a drug and a fluorescent probe for bioimaging in theranostics. In many cases, CNMs can also act as photothermal and photodynamic agents, besides being drug delivery vehicles, in order to enhance the effectiveness of cancer therapies [157].

Having targeted drug delivery is very important to enhance the effect of drugs and reduce the side effects caused by chemotherapeutics at physiological rather than pathological sites. Differences in microenvironmental conditions in cancer cells, especially cancer stem cells relative to normal cells, are often exploited to this end [158]. In this work, we discuss the last 5 years of progress in using CNMs as drug delivery agents, with a focus on molecular targeting (Table 4).

### 5.1. Fullerenes

Fullerene is a quasi-spherical and very hydrophobic carbon nanoparticle made of carbon atoms, with C_60_ being the most common. Due to its hydrophobic nature, it is a promising drug carrier to cross the BBB. For example, in a recent work, C_60_ was functionalized with the KLVFF peptide to specifically target the amyloid-β peptide involved in Alzheimer’s disease [159]. In this case, fullerenes were bound to an upconversion nanoparticle and acted as therapeutic agents, thanks to their ability to produce radical oxygen species (ROS) under near-infrared (NIR) light irradiation to prevent amyloid fibrillation and to act as radical scavengers in the dark and reduce the side effects of this approach.

**Figure 3:**
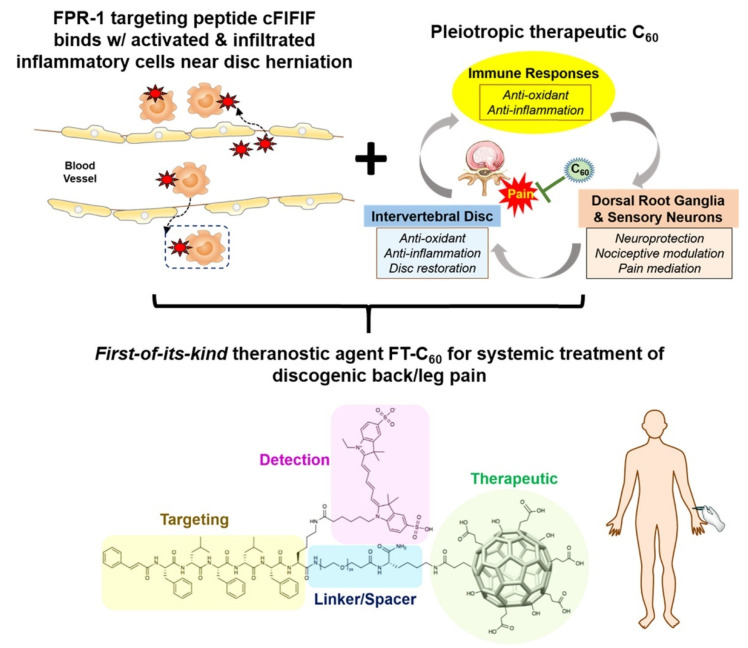
Schematic illustration of the therapeutic and targeting mechanism of the FIFIFK-functionalized C_60_ to treat discogenic pain. Reprinted with permission from [160]. Copyright © 2022, American Chemical Society.

However, the hydrophobic nature of fullerenes can be a problem for biological applications because they tend to aggregate and accumulate in organs such as liver, heart, and adrenal glands, potentially leading to toxicity [192]. For this reason, fullerenes are often functionalized with hydrophilic groups or molecules to increase their dispersibility in water. A theranostic agent based on a water-dispersible fullerene was developed to treat disc diseases, which are notably difficult therapeutic targets [160]. Fullerene was functionalized with hydroxyl and succinyl groups and bound to cyanine-5 for imaging and to the FIFIFK peptide to target the formyl peptide receptor 1 (FPR-1) specifically expressed in activated macrophages/monocytes under inflammatory insult. Fullerene acted as a radical scavenger and successfully inhibited inflammation, leading to pain alleviation in a mice model (Figure 3).

Fullerenols are polyhydroxy fullerenes that present fibrinolytic activity that is enhanced using a biomimetic carrier based on platelet and red-blood-cell membranes [161]. This approach has led to prolonged circulation time, better biocompatibility without toxic effects, and an enhanced affinity for fibrin due to the membrane proteins of the carrier. Finally, an innovative dual-responsive nanovehicle for the targeted delivery of the anticancer drug doxorubicin (DOX) has been developed [162]. Briefly, fullerene was non-covalently functionalized with the cationic and magnetic surfactant hexadecyltrimethylammonium trichloromonobromoferrate (CTAF), and this allowed the electrostatic interaction with anionic DNA to obtain a compact histone-like structure. Then, the complex was enveloped in a disulfide-modified hyaluronic acid (HA) shield. DNA served as an electrostatic scaffold to load DOX, while the disulfide-HA acted as both a redox trigger for drug delivery and a targeting vector due to the affinity of HA for the CD44 receptor that is overexpressed in many cancer cells.

### 5.2. CNOs

CNOs are multi-layered fullerenes, so they share some of their properties. However, CNOs can have different structures, for example, a more spherical or polyhedral shape or different numbers of layers. CNOs show very low toxicity and, differing from fullerenes, good light emission properties, so they have been studied as bioimaging probes [193].

**Figure 4:**
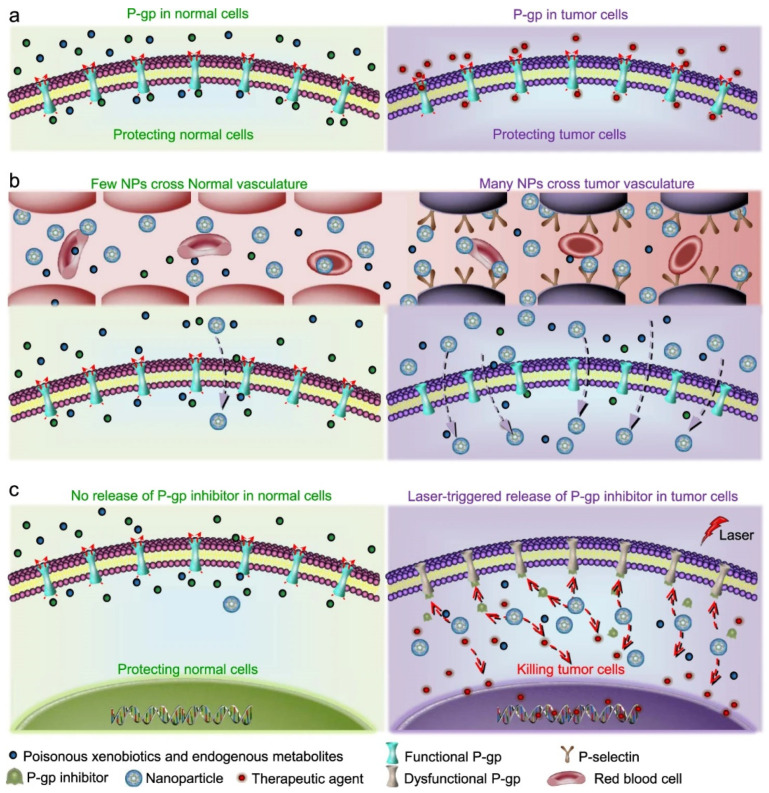
Schematic illustration of the strategy for targeting multi-drug resistant cancer cells and inhibiting the P-gp pump using CNOs. Reproduced from [163] under a Creative Commons license.

A recent work reported a strategy to overcome drug resistance in cancer based on blocking P-glycoprotein (P-gp), an ATP-binding membrane protein involved in the cellular efflux of many drugs that is overexpressed in multi-drug resistant cancer cells [163]. The result was achieved by co-delivering a P-gp inhibitor (HM30181A) and DOX using silica-CNO nanoparticles coated with fucoidan. While silica rendered CNOs more water-dispersible, selectivity was ensured by the affinity of fucoidan for P-selectin and a NIR-induced drug release (Figure 4).

### 5.3. CNTs

CNTs have excellent conductivity and anisotropic morphology; they are ideally suited for the stimulation of nerve [194,195,196] and cardiac [197,198,199] cells but also have the stigma of being associated with asbestos fibers’ toxicity [200], thus limiting their biological use. This has posed many limits to their clinical implementation, and, in 2019, they were also added to the ChemSec SIN list of nanomaterials of very high concern [201]. However, their toxicity strongly depends on a plethora of factors, including their functionalization, length, and purity, and grouping them all together is unjustified [53].

Recent works have reported the functionalization of CNTs with folic acid (FA) to target cancer cells overexpressing the FA receptor [164,165,166,167]. For example, a dual therapeutic agent was obtained by firstly oxidating CNTs to make them more water-dispersible and coating them with a biocompatible poly(*N*-vinyl pyrrole) polymer to enhance their photothermal properties. After that, FA was covalently linked to the composite, and the final material was impregnated with DOX to obtain a combined chemo-photothermal treatment with fewer side effects than free DOX [164]. A similar result was obtained with oxidized CNTs covered with bovine serum albumin as a nucleation site for growing gold nanoparticles to enhance their photothermal properties. Then, FA was linked to the gold nanoparticles via an S-functionalized PEG linker to ensure selective targeting, and DOX was adsorbed on the surface of the material [165]. A similar targeting approach was used to selectively deliver docetaxel and coumarin-6 to lung cancer cells using oxidized CNTs coated with FA-functionalized chitosan [167]. Finally, with a more sophisticated approach, an injectable hydrogel based on silk proteins and oxidized CNTs was developed for the NIR-triggered delivery of DOX to cancer cells overexpressing the FA receptor [166]. Oxidized CNTs were covalently linked to FA, and DOX was adsorbed on their surface; then, a silk-CNT hydrogel was formed. These recent examples manifest the increasing interest in the combination of CNTs with hydrogel biomaterials for therapeutic use and controlled delivery kinetics of bioactives [202].

DOX targeting of prostate cancer cells was achieved by simply using oxidized CNTs that were non-covalently functionalized with prostate-homing peptides and the drug [168]. Another cell-penetrating peptide, BR2, which is highly specific for cancer cells, was used for the selective co-delivery of DOX and surviving siRNA (short-interfering RNA) [169]. Oxidized CNTs were covalently linked to the targeting peptide and to betaine-modified polyethylenimine (PEI) to bind siRNA through electrostatic interactions and allow its pH-responsive endosomal escape. Betaine was necessary to reduce the side effects of PEI. The delivery of siRNA with cancer drugs is a novel gene therapeutic strategy to increase the effectiveness of cancer treatments. Another similar example was reported for the delivery of MBD1 siRNA using oxidized CNTs functionalized with a PEG-modified LyP-1 cyclic peptide, which bound specifically to p32 proteins overexpressed in pancreatic cancer cells [170]. CNTs were also covalently functionalized with PEI to bind a plasmid containing MBD1 siRNA and the gene encoding green fluorescent protein (GFP) for theranostics.

Another strategy for molecular targeting is the use of DNA sequences. A recent work reported the delivery of 5-fluoruracil (5-FU) together with an aptamer-siRNA chimera [171]. In this case, the targeting molecule was the AS1411 aptamer, a DNA sequence with anticancer properties for the specific binding of nucleolin, which is overexpressed in some cancer cells. This approach was studied as a treatment for the peritoneal dissemination of gastric cancer, which is known to be very difficult to treat. In another recent work, a more complex DNA-based targeting was achieved by the formation of a hydrogel based on DNA, silica nanoparticles, and CNTs for the more efficient delivery of DOX [172]. In this case, selective targeting was achieved by enriching DNA with CG-GC and aptamer sequences, and DOX was loaded through intercalation in the DNA scaffold. The hydrogel with CNTs was proven to be more cytotoxic for cancer cells compared to the gel without CNTs.

In another work, candesartan was used as both a drug and a targeting molecule as it has a strong affinity and inhibition ability for the angiotensin II type-1 receptor (AT1R) expressed on the surface of neovascular endothelial cells and many tumor cells [173]. Oxidized CNTs were functionalized with PEI as a linker, and candesartan was covalently bound to the linker. PEI also acted as a cationic scaffold to bind vascular endothelial growth factor (VEGF) siRNA, and the co-delivery of candesartan and VEGF siRNA provided a strong inhibition of tumor vascularization.

**Figure 5:**
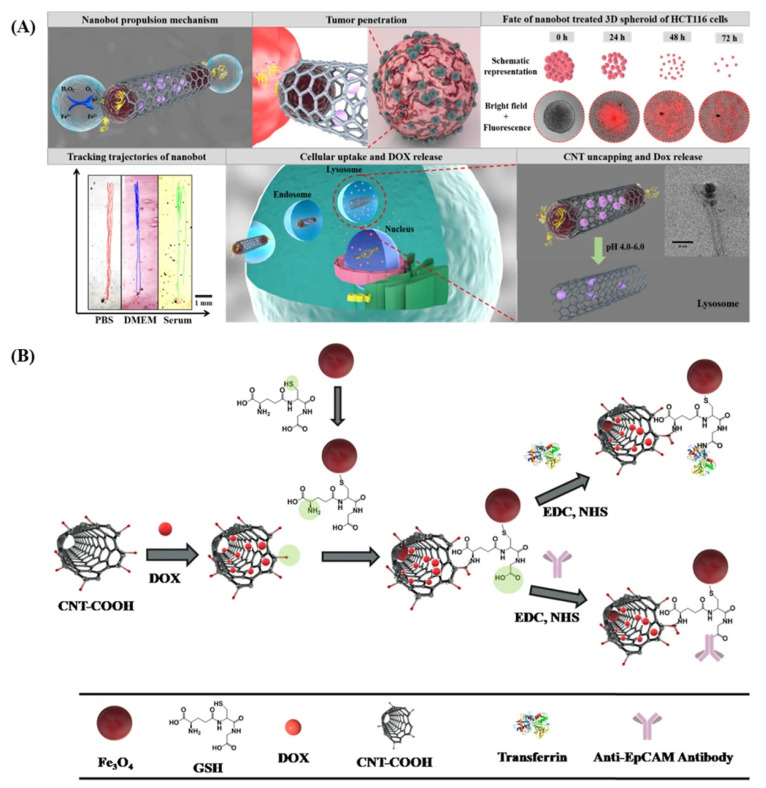
(**A**) Schematic representation of the oxygen-induced autonomous propulsion mechanism and deep penetration of nanobots in the tumor, the fate of 3D spheroids treated with nanobots, trajectories of nanobots in physiologically relevant media, followed by an illustration of the DOX-loaded nanobot targeting the transferrin/EpCAM receptor and entry into cancer cells, and, finally, the mechanism of triggered drug release under acidic endo/lysosomal conditions. (**B**) Schematic illustration of the step-by-step synthesis of CNT-DOX-Fe_3_O_4_-Tf/CNT-DOX-Fe_3_O_4_-mAb. Reproduced from [174], under a Creative Commons licence.

Finally, a novel and smart nanobot based on CNTs for the highly selective and fine-controlled delivery of DOX has been reported [174]. Briefly, DOX is encapsulated inside oxidized CNTs, and both ends are capped with Fe_3_O_4_ nanoparticles via a glutathione linker bound to CNTs with an acid-sensitive amide bond. The same glutathione linker is also used to functionalize CNTs with transferrin or an anti-epithelial cell adhesion molecule antibody (anti-EpCAM mAb) as a targeting vector for cancer cells. Fe_3_O_4_ nanoparticles allow the magnetic control of the bot and also self-propulsion due to the oxygen produced by the decomposition of the H_2_O_2_ naturally present in the human body, especially in the cancer environment, when catalyzed by the same nanoparticles. The acidic pH of lysosomes cleaves the amide bond linking CNTs and glutathione, freeing the CNTs’ end from the metal nanoparticles and allowing the specific release of DOX in cancer cells (Figure 5).

### 5.4. GQDs

GQDs are a new class of CNMs that is particularly known for their light emission properties, suitable for bioimaging. This feature, combined with their CNM nature and wide possibilities of functionalization, makes GQDs an ideal theranostic agent for drug delivery and bioimaging.

As an example, nitrogen-doped GQDs synthetized with a hydrothermal method from citric acid and urea were non-covalently loaded with methotrexate (MTX) [175]. This anticancer drug acts as a targeting agent as well due to its affinity for FA receptors, and MTT assays suggest that MTX-loaded GQDs are highly biocompatible and more effective than free MTX.

FA-functionalized GQDs were used for the targeted delivery of IR780 iodide, an effective photothermal agent and bioimaging probe [176]. However, the rigidity of the IR780 iodide molecule rendered it very insoluble in biocompatible solvents, while the loading on GQDs via π-π stacking interactions with the graphitic sp^2^ structure increased water solubility by over 2400-fold and the so-obtained composite resulted in an efficient theranostic platform for NIR fluorescence imaging and photothermal therapy. The FA-based targeting strategy was also used for the co-delivery of NO and a Pt(IV) prodrug [177]. In this case, the delivery platform was a nitrogen-doped GQD decorated with FA and a dinuclear Ru-Pt complex in which Ru(VI) acted as the NO carrier and Pt(IV) as a prodrug of a Pt(II) drug. NIR irradiation induced the release of NO and the Pt(IV) prodrug that was readily reduced to a Pt(II) active drug in the tumor environment, and the combination with the photothermal properties of GQDs produced a very synergic therapeutic agent.

A smart nano-antibiotic was produced using sulfur-doped GQDs (SGQDs) covalently linked to a CO-releasing molecule (CORM-401) and then electrostatically adsorbed with HA [178]. Secreted bacterial hyaluronidase damaged the HA shield, allowing GQD–CORM to penetrate bacterial cells. Then, under white light irradiation, the GQDs acted as ROS producers, and the subsequent release of CO-induced membrane and metabolic damage led to bacterial death. This novel combined photodynamic/CO gas therapy has been proven to be very safe for somatic cells and non-targeted bacteria and represents a new solution to overcome antibiotic resistance, besides being a diagnostic device for bacterial infections based on GQD fluorescence.

Another utilization of GQDs as theranostic agents was reported for the selective delivery of cisplatin using GQDs linked covalently to a single-chain variable fragment of an antibody that was specifically engineered to bind epidermal growth factor receptor (scFvB10) [179]. The molecular targeting, combined with the pH-dependent release of cisplatin, yielded a very selective nanocarrier that allowed the avoidance of the systematic side effects typical of cisplatin. In another work, a similar therapeutic strategy was reported using a GE11 peptide to target the VEGF receptor and simultaneously deliver cisplatin and DOX for the selective and synergic treatment of nasopharyngeal carcinoma [180].

**Figure 6:**
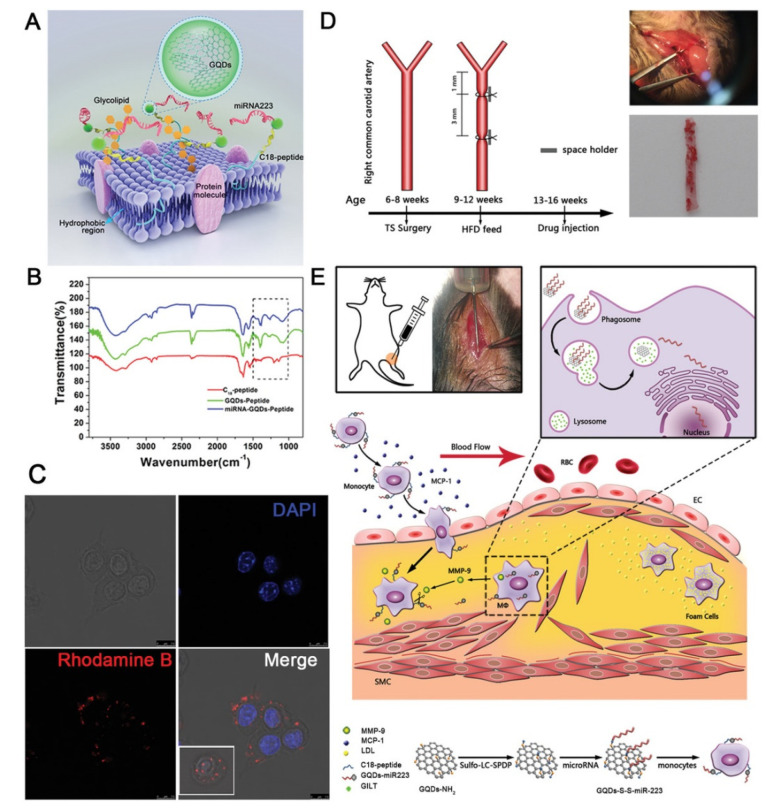
(**A**) Structure of the surface-engineered monocyte. (**B**) FT-IR spectra of C18P, GQDs-C18P, and miRNA223-GQDs-C18P, respectively. (**C**) Confocal fluorescence images of the surface-engineered monocyte. (**D**) Animal modeling process: partial ligation of the RCCA in *Apoe*^−/−^ mice 6–8 weeks of age; continued feeding with a high-fat diet for the 4 weeks after surgery; femoral vein injection after feeding with a high-fat diet for 4 weeks. Top, optical photograph of the surgery; bottom, microscopic image of the oil-red-O-positive area. Scale bar = 100 μm. (**E**) Delivery mechanism of monocyte-C18P-GQDs-miR223 in vivo. Reproduced with permission from [181]. © 2022 WILEY-VCH Verlag GmbH & Co. KGaA, Weinheim.

Finally, a novel biomimetic treatment for atherosclerotic diseases has been developed (Figure 6) [181]. Briefly, monocytes were used as selective carriers to target macrophages in the atherosclerotic plaque, and their membrane was engineered with a C18GVFHQTVSR peptide (C18P). This carefully designed peptide displayed a hydrophobic terminus that could be digested by matrix metalloproteinase-9 (MMP-9), which is overexpressed by macrophages in atherosclerotic plaque, and a hydrophobic C_18_ chain that was inserted in the cell membrane of the carrier monocyte. The free amino group of the peptide was linked to GQDs via an amide bond, and miRNA223 was linked to GQDs via a disulfide bond. The biomimetic carrier could successfully reach the atherosclerotic plaque as monocytes were continuously recruited there, and, after the selective cleavage of the C18P by MMP-9, GQD-miRNA entered the macrophages. The disulfide bond was then cleaved by *γ*-interferon-inducible lysosomal thiol reductase in the lysosomes, and miRNA223 was released, with the successful suppression of inflammatory pathways that lead to the formation of atherosclerotic plaque. This kind of smart approach can represent a valid alternative to the currently used statins, which are not completely effective in preventing ischemic strokes due to non-specific distribution.

### 5.5. Graphene-Based 2D Materials

Graphene-based 2D materials are a wide class of CNMs that include, for example, graphene, graphene oxide (GO), and reduced graphene oxide (rGO). They have attracted scientists’ attention in biomedicine because of their electronic properties, especially in the field of biosensing [203]; they are also very interesting for drug delivery due to their biocompatibility and wide possibilities for covalent or non-covalent functionalization.

**Figure 7:**
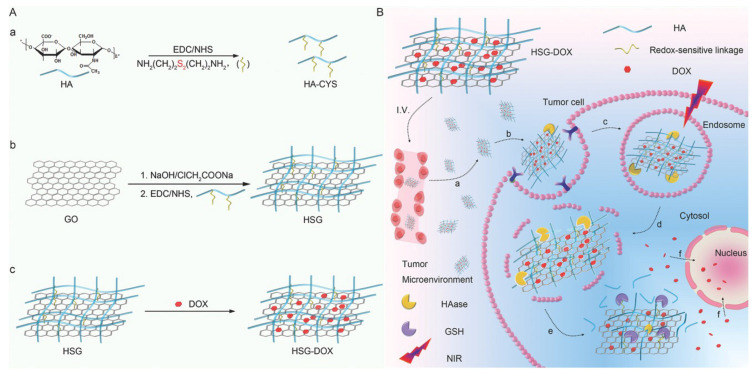
(**A**) Synthesis of bioreducible HA-GO-DOX (HSG-DOX) nanosheets. (**a**,**b**) HA is bound to graphene oxide (GO) using cystamine as a redox-sensitive linker or adipic dihydrazide as a redox-insensitive linker. (**c**) DOX loading on the so-modified graphene. (**B**) NIR-controlled endo/lysosomal escape and rapid release of DOX in cytoplasm induced by glutathione: (**a**) accumulation of HSG-DOX within the tumor site through passive and active targeting; (**b**,**c**) receptor-mediated cellular internalization; (**d**) hyaluronidase-mediated HA degradation in endosomes and NIR-mediated endo/lysosomal escape; (**e**) GSH triggered HA detachment and rapid DOX release in cytoplasm; (**f**) accumulation of DOX in nucleus for DNA damage-mediated apoptosis and cytotoxicity. Reproduced with permission from [182]. © 2022 WILEY-VCH Verlag GmbH & Co. KGaA, Weinheim.

A redox-responsive DOX carrier based on GO nanosheets was recently reported [182]. Briefly, GO nanosheets were functionalized with cystamine-modified HA through the free amino group of cystamine, and DOX was adsorbed on the material. The nanocarrier entered cancer cells via the CD44 receptor due to the presence of HA, and it was trapped in lysosomes, but a NIR trigger disrupted them thanks to the photothermal effect of GO nanosheets. Finally, the high concentration of glutathione in cancer cells’ cytoplasm cleaved the disulfide bond, and DOX was released, thanks to a NIR trigger, resulting in fine spatiotemporally controlled selectivity and an enhanced effect (Figure 7). In another recent work, DOX was successfully delivered to cancer cells with high selectivity using a composite material based on rGO functionalized with polydopamine to enhance the photothermal effect of rGO, then coated with mesoporous silica to increase DOX loading, and, finally, covered with HA to increase biocompatibility and ensure the selectivity of NIR-induced drug delivery to CD44 overexpressing cancer cells [183].

GO was also used for the molecular-targeted delivery of camptothecine by functionalization with an FA-bound PEG that guaranteed the desired selectivity for cancer cells [184]. However, the drug was only adsorbed on the material’s surface, and the release without any other trigger was too slow, especially in the acidic pH typical of cancer cells. Another recent work solved this problem with a redox-sensitive delivery system for DOX based on GO that was non-covalently functionalized with PEG-FA as the targeting molecule and with PEG-PLGA linked to DOX via a disulfide bond [185]. PEG polymers improved GO biocompatibility and stability in water, while the glutathione-sensitive disulfide bond increased the release rate and selectivity of DOX from GO. Cytotoxicity was higher for cancer cells and lower for normal cells compared to the free drug and non-targeted carriers.

A targeted carrier for the delivery of DOX was obtained with the functionalization of rGO with a cyclic RGDfC peptide via the thiol-maleimide click reaction [186]. The carrier alone showed an IC_50_ similar to free DOX in HeLa cells due to the too-slow release of the drug from rGO, but the use of a NIR trigger increased the release rate and improved the IC_50_, thanks to the photothermal properties of rGO, which also helped in killing cancer cells. A similar combined approach was developed to deliver berberine derivatives to cancer cells using AS1411-bound GO nanosheets as the targeted carrier [187].

A recent work reported another combined DOX/NIR approach with a smarter theranostic agent [188]. The carrier was based on GO functionalized with Fe_3_O_4_ and Cetuximab (CET), an anti-epidermal growth factor receptor, for dual magnetic and molecular targeting. In particular, magnetic nanoparticles were synthetized by chemical co-precipitation directly on GO, and carboxylic groups of the CNM were functionalized with avidin via an amidation reaction. Then the free biotin-binding sites on avidin were exploited to strongly bind biotin–PEG–CET for targeting and biotin–PEG–QD for bioimaging.

Another dual-targeted approach was developed to treat glioma with DOX, combining a magnetic targeting Fe_3_O_4_ and molecular brain targeting using transferrin, whose receptor is overexpressed on glioma cells and on the vascular endothelial cells of the BBB [189]. In this case, transferrin was bound to the magnetic nanoparticles, and this targeting strategy proved to be a promising solution to overcome the great obstacle represented by the BBB. Similar transferrin-based brain targeting was used to deliver puerarin to treat Parkinson’s disease (PD) [190]. This nanocarrier was tested in vivo on mice, and it gave excellent results in limiting PD-induced neuronal damage and also acted as a good antioxidant.

Finally, a novel dual gene therapy was studied for the treatment of pancreatic cancer, which is one of the most lethal types of cancer [191]. GO functionalized with FA as the targeting molecule was used as a carrier to deliver HDAC 1 siRNA and G12C mutant K-Ras siRNA together to pancreatic cancer cells, with a NIR-triggered release. The silencing of the two genes led to a strong inhibition in tumor cells’ proliferation without side effects on normal cells as selective targeting was guaranteed by the presence of FA and G12C mutant K-Ras siRNA, which is a mutation that is typical of pancreatic cancer cells.

## 6. Conclusions

CNMs have attracted wide interest in the scientific community for their engineering and application as drug delivery agents, thanks to their high surface area and drug-loading capacity, as well as ease of functionalization for active targeting of the pathological site and unique physico-chemical properties that enable bioimaging and photodynamic therapy. Despite their promising performance in vitro and in animal models, there are still many challenges to overcome for their clinical implementation, such as long-term toxicity assessments, cost-effective and sustainable production on a large scale, and the development of unified standards for their commercialization and correct classification. Nevertheless, the inclusion of targeting agents on their surface for the selective delivery of bioactive compounds to the pathological site has proven to be a very promising strategy thus far, as it has enabled the lowering of the dose of chemotherapeutics, with consequently reduced side effects. Clearly, further opportunities for development lie in personalized medicine, enabled by the biomarker profiling of patients’ samples. Furthermore, the ability of CNMs to cross the BBB is another key advantage in treating neurological disorders and neurodegenerative diseases; besides cancer, there has been recent interest in the use of CNMs as innovative therapeutic agents. To this end, their engineering to enable biodegradation or excretion appears to be a research area offering ample opportunities for development to enable the full unlocking of CNM potential to provide new solutions to unmet clinical needs.

## Figures and Tables

**Table 1:** Graphene-related standards that are publicly available. Reproduced from [56]; copyright © 2022, with permission from Elsevier. Data updated to February 2020.

Source	Tile of Standard	Publication Organization	Standard Number	Classification	Status
International	Nanotechnologies—Vocabulary—Part 13: Graphene and related two-dimensional (2D) materials	International Organization for Standardization (ISO)	ISO/TS 80004-13:2017	Terminology	Accept payment https://www.iso.org/standard/64741.html accessed on 21 May 2022
	Nanotechnologies—Matrix of properties and measurement techniques for graphene and related two-dimensional (2D) materials		ISO/TR 19733:2019	Measurement/ Method	Accept payment https://www.iso.org/standard/66188.html accessed on 21 May 2022
	Nanomanufacturing—Key control characteristics—Part 6–4: Graphene—Surface conductance measurement using resonant cavity	International Electrotechnical Commission (IEC)	IEC TS 62607-6-4:2016	Measurement/ Method	Accept payment https://webstore.iec.ch/publication/25950 accessed on 21 May 2022
China/ national	Nanotechnologies—Vocabulary—Part 13: Graphene and related two-dimensional (2D) materials	National Technical Committee 279 on Nanotechnology of Standardization Administration of China	GB/T 30544.13-2018	Terminology	Accept payment https://www.chinesestandard.net/China/Chinese.aspx/GBT30544.13-2018 accessed on 21 May 2022
	Nanotechnologies—Determination of specific surface area of graphene materials—Methylene blue adsorption method		GB/Z 38062-2019	Measurement/ Method	Open http://www.cssn.net.cn/cssn/front/110526250.html accessed on 21 May 2022
	Nanotechnologies—Quantitative analysis of the surface oxygen functional groups on graphene materials—Chemical titration method		GB/T 38114-2019	Measurement/ Method	Open http://www.gb688.cn/bzgk/gb/newGbInfo?hcno=05223E5FA0DF26920BA548B964F0928E accessed on 21 May 2022
	Graphene zinc coatings	Ministry of Industry and Information Technology, China	HG/T 5573-2019	Application	Open http://std.samr.gov.cn/hb/search/stdHBDetailed?id=9F25957A194447DAE05397BE0A0A0983 accessed on 21 May 2022
China/ associations	Graphene-enhanced extreme pressure lithium grease for construction machinery	Zhongguancun Standardization Association, Beijing, China	T/ZSA 74-2019	Application	Accept payment http://www.ttbz.org.cn/StandardManage/BuyDetail/32676/ accessed on 21 May 2022
	Graphene-modified rigid electric heating pad		T/ZSA 73-2019	Application	Accept payment http://www.ttbz.org.cn/StandardManage/BuyDetail/32675/ accessed on 21 May 2022
	Graphene-modified flexible electric heating film		T/ZSA 9001.01-2017	Application	Private http://www.ttbz.org.cn/StandardManage/Detail/21754/ accessed on 21 May 2022
	Epoxy graphene zinc primer	China Coating Industry Association	T/CNCIA 01003-2017	Application	Open http://www.ttbz.org.cn/StandardManage/Detail/22146/ accessed on 21 May 2022
	Waterborne graphene electromagnetic shielding coating for architecture		T/CNCIA 01004-2017	Application	Open http://www.ttbz.org.cn/StandardManage/Detail/22147/ accessed on 21 May 2022
	Graphene heating tiles	Guangdong Enterprise Innovation and Development Association, Guangdong Province, China	T/GDID 1012-2019	Application	Private http://www.ttbz.org.cn/StandardManage/Detail/32376/ accessed on 21 May 2022
	Graphene hollow yarn fabric with antibacterial and deodorant	Nantong textile industry association, Jiangsu Province, China	T/NTTIC 022-2019	Application	Private http://www.ttbz.org.cn/StandardManage/Detail/32223/ accessed on 21 May 2022
	Graphene materials terminology and designation	Zhongguancun Huaqing Innovation Alliance of the Graphene Industry, Beijing, China	T/CGIA 001-2018	Terminology	Accept payment http://www.ttbz.org.cn/StandardManage/BuyDetail/23102/ accessed on 21 May 2022
	Determination of silicon content in graphene materials—Molybdenum blue spectrophotometry		T/CGIA 013-2019	Measurement/ Method	Accept payment http://www.ttbz.org.cn/StandardManage/BuyDetail/30289/ accessed on 21 May 2022
	Determination of metallic elements in graphene materials—Inductively coupled plasma emission spectrometry		T/CGIA 012-2019	Measurement/ Method	Accept payment http://www.ttbz.org.cn/StandardManage/BuyDetail/30288/ accessed on 21 May 2022
	Test method of iodine adsorption number for graphene materials		T/CGIA 011-2019	Measurement/ Method	Accept payment http://www.ttbz.org.cn/StandardManage/Detail/29810/ accessed on 21 May 2022
	Guidance on naming of products containing graphene materials		T/CGIA 002-2018	Measurement/ Method	Accept payment http://www.ttbz.org.cn/StandardManage/BuyDetail/23101/ accessed on 21 May 2022
	Graphene-enhanced extreme pressure lithium grease for construction machinery		T/CGIA 31-2019	Application	Accept payment http://www.ttbz.org.cn/StandardManage/BuyDetail/30287/ accessed on 21 May 2022
	Graphene materials conductive suspension for use in lithium-ion battery application		T/CGIA 032-2019	Application	Private standard@c-gia.org
	Electric infrared radiant heating film made by printing ink-based graphene materials		T/CGIA 030-2017	Application	Private http://www.ttbz.org.cn/StandardManage/Detail/4045/ accessed on 21 May 2022
	Test method for identification of graphene materials in fibers—Transmission electron microscope (TEM) method	China National Textile and Apparel Council	T/CNTAC 21-2018	Measurement/ Method	Private http://www.ttbz.org.cn/StandardManage/Detail/25301/ accessed on 21 May 2022

**Table 2:** Recent examples of green methods to produce graphitic carbon nanostructures using green materials and/or lower energy-demanding processes.

Carbon Nanostructure	Green Process	Carbon Source	Applications	Ref.
CNOs	Pyrolysis	Flaxseed oil	Photocatalysis Al(III) detection	[71]
	Pyrolysis	Waste frying oil	Capacitors	[72]
	Microwave pyrolysis	Fish scale	LED	[73]
	Soxhlet purification	Pollutant soot	Cell imaging Cr(VI) detection Strain sensing Dyes removal	[74,75,76]
	Molten salt electrolysis	CO_2_	n.a.	[77,78]
	Candle burning	Candle soot	Cancer therapy Bioimaging Bisphenol A removal	[79,80]
	Hydrothermal	Citric acid	n.a.	[81]
	Catalyzed carbonization	Rice husk	Capacitors	[82]
	Ball milling	Graphite	n.a.	[83]
Fullerenes	Catalytic thermal decomposition	Plastic waste	Dyes removal	[84]
CNTs	Molten salt electrolysis	CO_2_	n.a.	[77,78,85,86,87,88,89,90,91,92,93,94,95,96,97]
	CVD	Barbeque grease	n.a.	[98]
	CVD	Plant extract	n.a.	[99]
	CVD	Flying ash	Lubricants	[100]
	CVD	Plastic waste	n.a.	[101,102,103,104,105,106,107,108,109,110,111,112,113,114,115]
	CVD	Plastic waste	Adsorption	[116]
	CVD	Plastic waste	Oxygen reduction	[117]
	Spray pyrolysis	Coconut and olive oil	n.a.	[118]
	Catalyzed pyrolysis	Plastic waste	Lubricants	[119]
GQDs	Electrochemistry	Graphite	Radioimaging	[120]
	Electrochemistry	Wood charcoal	Peroxidase mimic	[121]
	Gamma irradiation	Graphite	Photodynamic therapy	[122]
	Hydrothermal	Fruit	Bioimaging Ag(I) sensing	[123]
	Hydrothermal	Cotton	Bioimaging	[124]
	Hydrothermal	Starch	Bioimaging	[125]
	Hydrothermal	Lemon juice	n.a.	[126]
	Pyrolysis	Citric acid	Hg(II) sensing	[127]
	Pyrolysis	Casein	Hg(II) and thiols sensing Bioimaging	[128]
	Mild oxidation	Coal tar pitch	n.a.	[129]
	UV irradiation	Salicylic acid	Bioimaging	[130]
	Microwave	Grape seed	Bioimaging	[131]
Graphene-based 2D materials	Soxhlet purification	Pollutant soot	Dyes degradation	[132,133]
	Catalytic reduction	Graphene oxide	n.a.	[134]
	Reduction with plant extract	Graphene oxide	Ni(II) removal	[135]
	Chemical graphitization and reduction with ascorbic acid	Charcoal	Lubricants	[119]
	Thermal decomposition	Plastic waste	Dyes removal	[136,137]
	Pyrolysis and ball milling	Plastic waste	Energy storage	[138]
	CVD	Plastic waste	Electrodes	[139]
	CVD	CO_2_	n.a.	[140]
	Molten salt electrolysis	CO_2_	n.a.	[141]

**Table 3:** Recently reported processes for the CVD formation of CNTs from plastic waste. ^1^ LDPE = low-density PE. ^2^ MP = mixed plastics. ^3^ HDPE = high-density PE.

Plastic Polymer	Pyrolysis Temperature (°C)	CVD Temperature (°C)	Catalyst	Condensation Step	I_D_/I_G_	Ref.
PP	500	800	Fe-Ni	No	0.82	[101]
PP	500	700	Cu-Ni/La_2_O_3_	Yes	0.69	[102]
LDPE ^1^	400	750	Fe-Mo/MgO	Yes	0.51	[103]
PP	500	900	Stainless-steel	No	0.48	[104]
LDPE ^1^	600	700	Ni-Mo/Al_2_O_3_	Yes	0.93	[105]
LDPE ^1^	700	650	Ni-Mo/Al_2_O_3_	Yes	1.26	[106]
PP	700	650	Ni-Mo/Al_2_O_3_	Yes	1.31	[106]
MP ^2^	700	900	Ni-Mo/MgO	Yes	0.71	[107]
LDPE ^1^	700	800	Ni/La	Yes	0.47	[108]
PP	700	800	Ni/La	Yes	0.42	[108]
HDPE ^3^	500	700	Ni/AAO	No	n.a.	[109]
HDPE ^3^	500	700	Ni/ceramic	No	n.a.	[110]
PE	800	800	Stainless-steel	No	0.36	[111]
MP ^2^	500	800	Ni-Fe	No	0.52	[112,113]
LDPE ^1^, PP	450	800	Co/MgO	No	Low	[114]
LDPE ^1^	500	700	Co-Mo/MgO	Yes	0.70	[115]
LDPE ^1^, PP, MP ^2^	600	500	NiO/CaCO_3_	No	≈1.5	[117]
LDPE ^1^, PP, MP ^2^	600	800	NiO/CaCO_3_	No	≈0.4	[117]

**Table 4:** Recent examples of the use of CNMs in molecular-targeted drug delivery.

CNM Type	Therapeutic Agent	Targeting Agent	Disease	Ref.
Fullerene	Fullerene	KLVFF peptide	Alzheimer	[159]
	Fullerene	FIFIFK peptide	Disc diseases	[160]
	Fullerenol	Blood-cell membrane	Thrombotic disease	[161]
	Doxorubicin	Hyaluronic acid (HA)	Cancer	[162]
CNOs	Doxorubicin HM30181A	Fucoidan	Cancer	[163]
CNTs	Doxorubicin	Folic acid	Cancer	[164,165,166]
	Docetaxel Coumarin-6	Folic acid	Lung cancer	[167]
	Doxorubicin	Prostate-homing peptide	Prostate cancer	[168]
	Doxorubicin Survivin siRNA	BR_2_ peptide	Cancer	[169]
	MBD1 siRNA	LyP-1 peptide	Pancreatic cancer	[170]
	AS1411 aptamer 5-fluorouracil p38 MAPK siRNA	AS1411 aptamer	Peritoneal dissemination of gastric cancer	[171]
	Doxorubicin	DNA	Cancer	[172]
	VEGF siRNA Candesartan	Candesartan	Cancer	[173]
	Doxorubicin	Transferrin anti-EpCAM mAb	Cancer	[174]
GQDs	Methotrexate	Methotrexate	Cancer	[175]
	IR780 iodide	Folic acid	Cancer	[176]
	Pt(IV) prodrug NO	Folic acid	Cancer	[177]
	CO	Hyaluronic acid (HA)	Bacterial infections	[178]
	Cisplatin	scFvB10	Breast cancer	[179]
	Doxorubicin Cisplatin	GE11 peptide	Nasopharyngeal carcinoma	[180]
	miRNA223	Monocyte	Atherosclerosis	[181]
Graphene-based 2D materials	Doxorubicin	Hyaluronic acid (HA)	Cancer	[182,183]
	Camptothecin	Folic acid	Cancer	[184]
	Doxorubicin	Folic acid	Cancer	[185]
	Doxorubicin	RGDfC peptide	Cancer	[186]
	Berberine AS1411 aptamer	AS1411 aptamer	Nucleolin-positive cancer	[187]
	Doxorubicin	Cetuximab	Colon carcinoma	[188]
	Doxorubicin	Lactoferrin	Glioma	[189]
	Puerarin	Lactoferrin	Parkinson	[190]
	HDAC 1 siRNA K-Ras siRNA	Folic acid	Pancreatic cancer	[191]

## Data Availability

Not applicable.
